# Association between sodium glucose co-transporter 2 inhibitors and a reduced risk of heart failure in patients with type 2 diabetes mellitus: a real-world nationwide population-based cohort study

**DOI:** 10.1186/s12933-018-0737-5

**Published:** 2018-06-23

**Authors:** Young-Gun Kim, Seung Jin Han, Dae Jung Kim, Kwan-Woo Lee, Hae Jin Kim

**Affiliations:** 1 Department of Medical Sciences, Ajou University Graduate School, Suwon, Republic of Korea; 2 Department of Internal Medicine, Incheon Medical Center, Incheon, Republic of Korea; 3 Department of Endocrinology and Metabolism, Ajou University School of Medicine, Suwon, Republic of Korea

**Keywords:** Heart failure, Type 2 diabetes mellitus, Sodium glucose co-transporter 2 inhibitors dipeptidyl peptidase-4 inhibitor

## Abstract

**Background:**

Recently, two large randomized controlled trials which only included patients with underlying cardiovascular disease (CVD) or patients at high risk for CVD showed that two sodium glucose co-transporter 2 inhibitors (SGLT-2is) significantly reduced hospitalization for heart failure (hHF), with an early separation in the survival curves for hHF. There were concerns whether SGLT-2i use could protect hHF in patients without CVD and how soon SGLT-2i-treated patients show a lower risk of hHF. Thus, we aimed to evaluate whether the heart failure protective effect of SGLT-2i differs depending on the underlying CVD and the prescription period compared with dipeptidyl peptidase-4 inhibitors (DPP-4i).

**Methods:**

We performed a nationwide retrospective observational study to estimate the effect of SGLT-2i on HF. The 59,479 SGLT-2i new-users were matched with same number of DPP-4i new-users through propensity score matching using 53 confounding variables. Kaplan–Meier (K–M) curves and Cox proportional hazards regression analyses were used to estimate the risk of hospitalization for hHF.

**Results:**

The incidence rates of hHF were 0.83 and 1.13 per 100 person-years in SGLT-2i-treated patients and DPP-4i-treated patients, respectively. The hazard ratios of hHF were 0.66 (95% confidence interval 0.58–0.75) in SGLT-2i-treated patients compared with the DPP-4i-treated patients. Among the patients with underlying CVD, SGLT-2i-treated patients were associated with a lower risk of hHF from 30 days to 3 years after initiating drugs compared with DPP-4i. However, SGLT-2i use only showed a lower risk of hHF with a significant difference 3 years after drug initiation among patients without underlying CVD.

**Conclusions:**

Our findings suggest that SGLT-2i reduced hHF compared with DPP-4i. A heart failure protective effect of SGLT-2i use vs. DPP-4i use was shown 30 days after initiating the SGLT-2i among patients with established CVD, but this effect appeared later in patients without established CVD.

**Electronic supplementary material:**

The online version of this article (10.1186/s12933-018-0737-5) contains supplementary material, which is available to authorized users.

## Introduction

Heart failure (HF) occurs in 1–2% of the world’s population [1]. Despite standard therapy, such as renin–angiotensin–aldosterone system inhibitors, beta blockers, or diuretics, there is a requirement for an unmet need [2]. In particular, HF is a major comorbidity in patients with type 2 diabetes mellitus (T2DM), which is caused by diverse pathogenic factors such as the cardiotoxic tetrad of coronary artery disease, diabetic cardiomyopathy, or hypertension. HF reduces quality of life, results in hospitalization, and contributes to a large socioeconomic loss [1, 3].

Some studies have indicated that HF is associated with oral hypoglycemic agents, which has attracted worldwide attention. Rosiglitazone has been withdrawn from the market because it was reported to cause HF. Saxagliptin has been reported to be associate with HF, which has caused global controversy [4, 5]. Unlike these two drugs, empagliflozin reduced hospitalization for heart failure (hHF) by 35% in a randomized controlled trial (RCT) called the Empagliflozin Cardiovascular Outcome Event Trial in Type 2 Diabetes Mellitus Patients (EMPA-REG OUTCOME) [6], and this tendency was the same when only Asian patients were analyzed [7]. In another RCT, the Canagliflozin Cardiovascular Assessment Study (CANVAS) Program, canagliflozin reduced hHF, suggesting the protective effect of sodium glucose co-transporter 2 inhibitors (SGLT-2i) as a class effect on HF [8]. Although there is no RCT, multinational observational studies have shown that dapagliflozin is also protective against HF [9]. In the EMPA-REG OUTCOME trial, only patients with established cardiovascular disease (CVD) were included. About 70% of the participants in the CANVAS program had established CVD, and the remaining participants were patients with two or more CVD risks. Thus, after the results of these studies were published, concerns about the effect of SGLT-2i on HF in patients without established CVD were raised.

An early separation of survival curves for hHF was observed in the EMPA-REG OUTCOME trial and CANVAS Program, although no differences were observed in myocardial infarction or cerebral infarction [6, 8]. This finding suggests that the HF protective effect of SGLT-2i could be derived through a non-glycemic anti-atherosclerotic mechanism, such as a diuretic effect, a hemo-concentration effect, a ketone body-producing effect, or a uric acid lowering effect. However, to the best of our knowledge, no observational study has been conducted to determine how soon SGLT-2i-treated patients show a lower incidence of hHF.

We compared HF risk of SGLT-2i with dipeptidyl peptidase-4 inhibitors (DPP-4i) as an active comparator because both drugs are similarly used as a second-line treatment and have a low risk of hypoglycemia and weight gain, as well as a potent glucose-lowering effect. Although there is still debate and no consensus, DPP-4i has been shown not to elevate the risk of heart failure in many studies [10–13].

We aimed to evaluate the HF protective effect of SGLT-2i compared with DPP-4i. In addition, we sought to estimate whether the HF protective effect of SGLT-2i differs depending on the underlying CVD and the prescription period.

## Methods

### Study design and data source

We performed a nationwide retrospective observational study to estimate the effect of SGLT-2i on HF. The health records from the Korean Health Insurance Review and Assessment Service (HIRA) database were analyzed. All records were de-identified according to relevant laws and regulations. This database covers > 99% of the South Korean population and includes all health records, such as demographics, diagnoses (coded with International Classification of Diseases [ICD]-10), drug prescriptions, and procedures. We used the data from January 1, 2013 to June 30, 2017. Our study protocol was reviewed and approved by the Institutional Review Board (IRB) of Ajou University Hospital (AJIRB-MED-EXP-17-497), and informed consent was waived by the IRB.

### Patient cohort

**Fig. 1:**
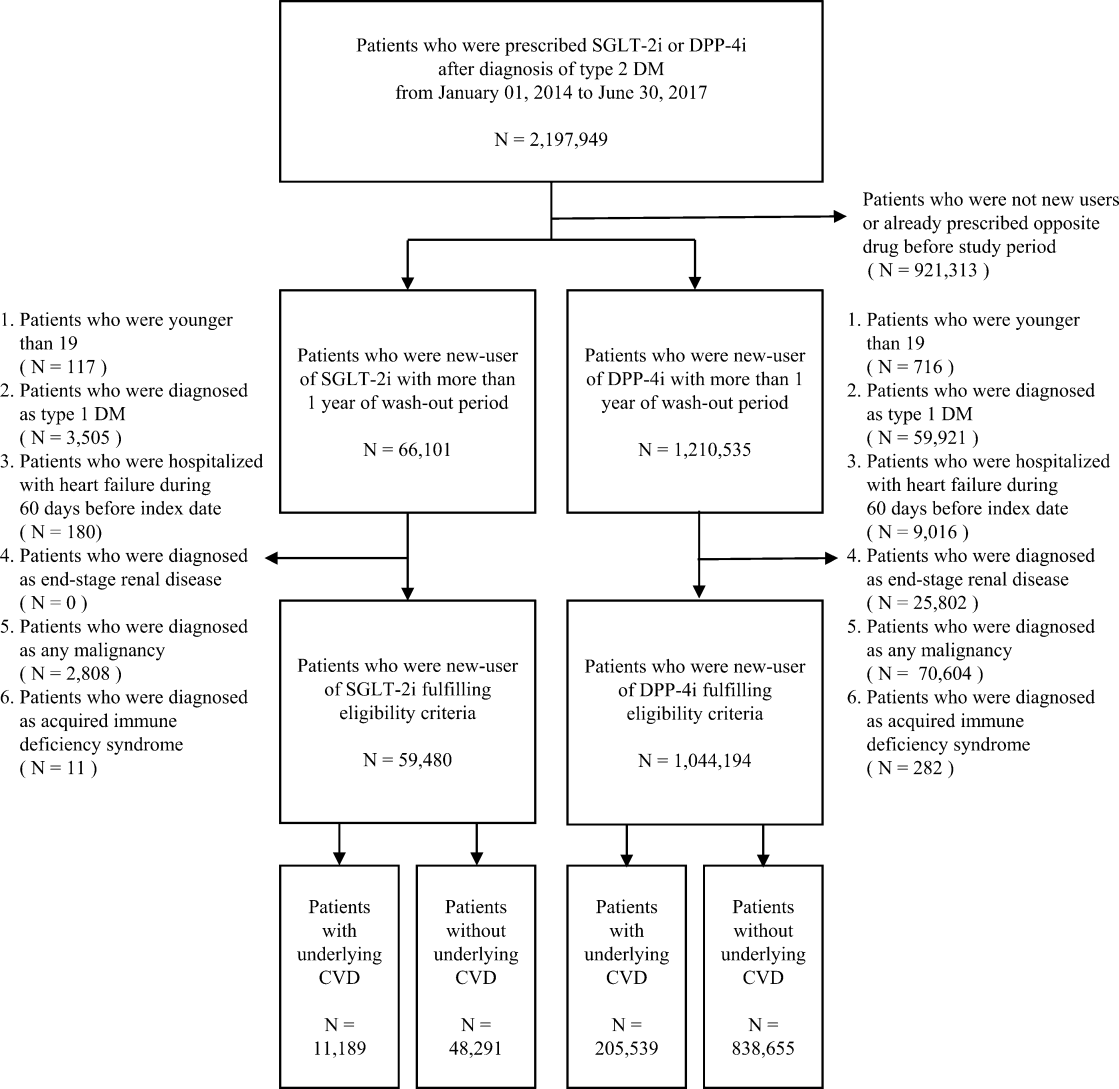
Flow chart of the sample selection, stratified by underlying cardiovascular disease. *CVD* cardiovascular disease, *DM* diabetes mellitus, *DPP-4i* dipeptidyl-peptidase IV inhibitor, *N* number, *SGLT-2i* sodium-glucose co-transporter 2 inhibitor

Patients aged > 19 years with T2DM (ICD-10 code: E11) who were new users of SGLT-2i or DPP-4i were included in the cohort. A new user was defined as a patient who had more than a 1-year wash-out period before the first SGLT-2i or DPP-4i prescription (The first index date was Jan 1, 2014). The first prescribed drug was defined as the index drug and the first prescription date was designated the index date. Patients who were diagnosed with type 1 diabetes mellitus, end-stage renal disease, acquired immune deficiency syndrome, any malignancy, or previous hHF 60 days before the index date were excluded. A flow chart is shown in Fig. 1.

### Outcomes

The study outcome was hHF (diagnosed as ICD-10 code I50 during the admission) after initiating SGLT-2i. The study cohort was stratified according to whether the patient had established CVD (diagnosed as HF, myocardial infarction, other ischemic heart disease, stroke, cerebrovascular disease, peripheral artery occlusive disease with an ICD-10 code, or received percutaneous coronary intervention or a coronary artery bypass graft). To evaluate whether the HF risk of SGLT-2i varied with the follow-up period after the time of initiation, analyses were performed according to the time after initiation of the drug (30, 90, 180 days, 1, and 3 years after the index date) in all patients and each CVD stratum.

### Statistical analysis

All analyses were performed with SAS (ver. 9.4; SAS Institute, Cary, NC, USA) and R software (ver. 3.4.1; R Development Core Team, Vienna, Austria). All values are presented as mean ± standard deviation (SD). To minimize differences in the baseline characteristics between the SGLT-2i and DPP-4i groups, propensity score matching was performed with 53 variables which were presented in Table 1 (sex, age, underlying disease [1 year prior to the index date], prescribed drugs [180 days prior to the index date, particularly beta-blockers, angiotensin converting enzyme inhibitors, angiotensin receptor blockers, aldosterone antagonists, loop diuretics, and thiazides, which may affect hospitalization for HF, were also included], cardiologist visits [30 days prior to the index date], hospitalization [30 or 31–365 days prior to the index date], emergency department visit [365 days prior to the index date]). The nearest neighbor matching was used with a caliper (0.1). Propensity score matching was performed three times (DPP-4i group vs. SGLT-2i group in all patients, and CVD stratification) with a 1:1 ratio. Differences between the two groups were calculated with standardized differences and absolute values < 0.1 (10%) of standardized differences were considered to be no difference.

**Table 1:** Baseline characteristics of matched pairs in all patients

	DPP-4i (n = 59,479)	SGLT-2i (n = 59,479)	Standardized difference
Age (years), mean (SD)	53.4 (12.4)	53.2 (11.9)	0.012
Male	54.75	54.86	0.002
Hypertension	60.46	60.33	0.003
Dyslipidemia	81.98	82.05	0.002
Chronic kidney disease	7.74	7.55	0.007
Cardiovascular disease			
AMI	1.57	1.43	0.011
Other ischemic heart disease	14.25	14.02	0.007
Heart failure	3.39	3.41	0.001
Cerebral infarction	3.82	3.71	0.006
Cerebrovascular event	5.33	5.24	0.004
Peripheral artery occlusive disease	0.66	0.61	0.006
Coronary revascularization procedures			
Coronary artery bypass graft	0.04	0.03	0.003
Percutaneous coronary intervention	2.02	1.99	0.002
Microvascular complications of diabetes			
Nephropathy	10.46	10.34	0.004
Neuropathy	7.17	6.99	0.007
Retinopathy	13.04	12.93	0.003
Atrial fibrillation	1.58	1.52	0.004
Other heart disease	11.79	11.71	0.002
Hypoglycemia	2.2	2.16	0.002
Asthma	12.56	12.49	0.002
Chronic obstructive pulmonary disease	4.84	4.74	0.005
Connective tissue disease	3.57	3.58	0.001
Pancreatitis	1.5	1.49	0.001
Osteoporosis	7.67	7.36	0.012
Alcohol intake^a^	4.66	4.65	<0.001
Smoking^a^	0.15	0.16	0.002
Obesity^a^	0.39	0.39	0.001
Medication use (180 days prior to index date)			
Antidiabetic agent			
Metformin	80.88	81.18	0.008
Sulfonylurea	37.29	36.77	0.011
Thiazolidinediones	9.74	9.11	0.022
Alpha-glucosidase inhibitor	4.86	4.71	0.007
Meglitinide	0.81	0.73	0.009
Insulin	14.45	13.84	0.017
Diuretics			
Loop diuretics	3.83	3.79	0.002
Thiazide	14.86	14.68	0.005
Aldosterone antagonist	2.01	1.92	0.006
Potassium sparing diuretics	0.05	0.06	0.006
Anti-hypertensive agent			
Calcium channel blocker	29.7	29.74	0.001
ACEI	3.06	2.95	0.007
ARB	47.68	47.62	0.001
Beta blocker	9.92	9.65	0.009
Alpha blocker	0.71	0.67	0.005
Digoxin	0.7	0.72	0.002
Aspirin	23.39	22.98	0.01
P2Y12 inhibitor	8.55	8.38	0.006
Warfarin	0.58	0.58	<0.001
NOAC	0.66	0.53	0.017
Lipid-lowering agent			
Statin	53.42	53.02	0.008
Fibrate	10.59	10.74	0.005
Ezetimibe	8.54	8.51	0.001
Cardiologist visit (30 days prior to index date)	12.47	12.4	0.002
Hospitalization (30 days prior to index date)	7.33	6.9	0.017
Hospitalization (30–365 days prior to index date)	20.96	20.3	0.016
Emergency department visit (365 days prior to index date)	6.08	5.84	0.010

Data presented as frequencies in percentage or means (SD)

Less than 0.1 (10%) on the absolute value of standardized difference was considered as a negligible difference between groups. The mean (SD) standardized difference of all covariates was 0.59% (0.49%)

*ACEI* angiotensin-converting-enzyme inhibitor, *AMI* acute myocardial infarction, *ARB* angiotensin II receptor antagonists, *DPP-4i* dipeptidyl-peptidase IV inhibitor, *NOAC* novel oral anticoagulant, *SD* standard deviation, *SGLT2i* sodium-glucose co-transporter 2 inhibitor, *SU* sulfonylurea

^a^Confirmed by diagnosis code (International Classification of Diseases, 10th revision)

After propensity score matching, we performed survival analyses to estimate the effect of SGLT-2i on hHF. The Kaplan–Meier estimates were performed on matched pairs using 1 minus the Kaplan–Meier estimate. Cox proportional hazards regressions were performed with matched pairs in all patients and each CVD stratum. To determine whether the hHF risk of SGLT-2i varied with the follow-up period after the time of initiation, analyses were performed according to the time after initiating the drug (30, 90, 180 days, 1, and 3 years after the index date).

## Results

### Study population and patient characteristics

A total of 1,103,674 new users were included in the cohort (1,044,194 new users in the DPP-4i group and 59,480 new users in the SGLT-2i group) and 1,128,528 person-years. In total, 59,479 pairs were included after propensity score matching. According to underlying CVD, 11,188 and 48,290 pairs were matched in patients with and without underlying CVD, respectively.

The baseline characteristics of all patients before propensity score matching are presented in Additional file 1: Table S1 and those of the matched patients are presented in Table 1 and Additional file 1: Tables S2 and S3. The standardized differences in all variables were < 0.1 (10%) and means (SD) of the standardized differences were 0.59% (0.49%), 1.01% (1.33%), and 0.96% (2.03%) in all patients and patients with and without CVD, respectively. Thus, the differences between matched pairs were statistically negligible. The mean follow-up period of the matched patients was 318.5 days. The SGLT2 inhibitor group consisted of dapagliflozin (90.2%) and ipragliflozin (9.8%) and the DPP-4 inhibitors group consisted of linagliptin (26.3%), sitagliptin (26.1%), gemigliptin (15.6%), vildagliptin (10.0%), alogliptin (7.8%), saxagliptin (5.4%), teneligliptin (5.2%), anagliptin (2.0%), and evogliptin (1.7%).

### Heart failure risk of SGLT-2i vs. DPP-4i

**Fig. 2:**
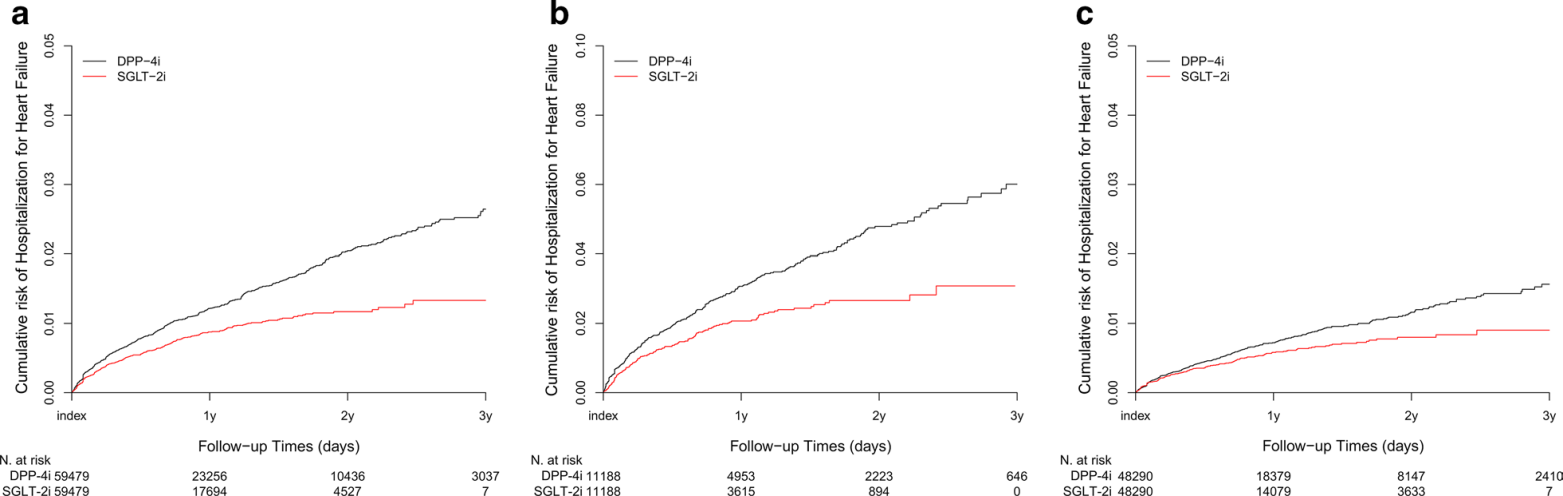
Kaplan–Meier plots of hospitalization for heart failure in all patients (**a**) and baseline cardiovascular stratifications (**b** with baseline cardiovascular disease, **c** without baseline cardiovascular disease). *DPP-4i* dipeptidyl-peptidase IV inhibitor, *N* number, *SGLT-2i* sodium-glucose co-transporter 2 inhibitor, *y* year(s)

In total, 1025 hHF events were observed among the matched patients during the follow-up period. The incidence rates of hHF were 0.83 and 1.13 per 100 person-years in SGLT-2i-treated patients and DPP-4i-treated patients, respectively. The hazard ratios of hHF were 0.66 (95% confidence interval [CI] 0.58–0.75, p < 0.001) in SGLT-2i-treated patients compared with the DPP-4i-treated patients (Fig. 2 and Table 2). SGLT-2i use showed a significantly lower risk of hHF at 30, 90, and 180 days, as well as at 1 and 3 years after the initiating the drug vs. DPP-4i use (Table 2). In addition, among the patients with underlying CVD, SGLT-2i-treated patients were associated with a lower risk of hHF across all time points compared with DPP-4i. However, SGLT-2i use only showed a lower risk of hHF with a significant difference 3 years after drug initiation compared with DPP-4i use among patients without underlying CVD.

**Table 2:** The risk of hospitalization of SGLT-2i treated patients for heart failure according to follow-up period compared with DPP-4i treated patients

Days after the drug initiation	Events	HR	lower CI	upper CI	p value
Total patients (N = 118,958)					
30 days	256	0.74	0.58	0.95	0.02
90 days	456	0.75	0.62	0.90	0.002
180 days	617	0.71	0.61	0.84	< 0.001
1 year	818	0.73	0.63	0.84	< 0.001
3 years	1025	0.66	0.58	0.75	< 0.001
Patients with underlying cardiovascular disease (N = 22,376)					
30 days	117	0.62	0.43	0.91	0.01
90 days	220	0.73	0.5666	0.95	0.02
180 days	296	0.69	0.55	0.88	< 0.001
1 year	400	0.69	0.56	0.84	< 0.001
3 years	492	0.66	0.58	0.78	< 0.001
Patients without underlying cardiovascular disease (N = 96,580)					
30 days	123	1.09	0.76	1.55	0.65
90 days	218	0.89	0.68	1.16	0.39
180 days	298	0.83	0.66	1.05	0.12
1 year	403	0.83	0.68	0.84	0.06
3 years	502	0.76	0.63	0.95	0.003

*CI* 95% confidence interval, *HR* hazard ratio; *N* number of patients

## Discussion

Our results show that SGLT-2i use reduced hHF compared with DPP-4i use. Patients with underlying CVD were associated with a reduced risk of hHF from 30 days after initiating the SGLT-2i to the third year, but patients without underlying CVD showed a significantly lower risk of HF in the third year.

In our study, SGLT-2i use reduced hHF by 36% compared with DPP-4i use, which is consistent with the results of the EMPA-REG OUTCOME trial, the CANVAS Program, and three observational studies [6, 8, 9, 14, 15]. In the EMPA-REG OUTCOME trial, empagliflozin reduced hHF by 35% (95% CI, 0.50–0.85; p = 0.002) vs. placebo [6]. In addition, in the CANVAS Program, which included data integrated from the CANVAS and CANVAS-R trials, canagliflozin showed a 33% reduction in hHF (95% CI, 0.52–0.87) vs. placebo [8]. The finding that hHF began to be separated widely within 3 months and was maintained for 3 years in two previous RCTs has brought worldwide interest [2, 16–18]. However, no statistical analysis has been performed to determine how soon patients benefit for hHF after using SGLT-2i; thus, there is a need to analyze the impact of hHF by dividing the duration of SGLT-2i use. Therefore, we analyzed hHF according to the duration of SGLT-2i use and showed that hHF was significantly lower in SGLT-2i new users vs. DPP-4i new users beginning 30 days after initiating SGLT-2i. This finding suggests that patients with T2DM and established CVD may benefit from HF management at the initiation of SGLT-2i use.

Several mechanisms have been proposed to explain the early HF protective effect of SGLT-2i. First, it induces a negative caloric balance and reduces hemoglobin A1c and body weight by causing glycosuria [16]. However, our results show that hHF decreased beginning 30 days after initiating the SGLT-2i, which was similar to previous RCTs [6, 8]. The protective effect of HF is achievable within a short time although improvements in the glycemic and lipid profiles were modest at 3 months in those RCTs; thus, it likely occurs via a non-glycemic mechanism. Second, SGLT-2i improves hemodynamics through natriuresis, which was suggested as a pivotal mechanism to improve HF in the early phase of drug initiation in some studies [2, 19–21]. Sodium glucose co-transporter 2 resorbs about 5% of the sodium under normal conditions and this capacity increases under a chronic hyperglycemic condition [2, 16, 22–24]. Thus, SGLT-2i decrease plasma volume which consequently reduces preload and ventricular filling pressure, and lowers blood pressure, which causes a reduction in afterload and improves subendocardial blood flow. Third, a shift in fuel energetics has been suggested as a protective mechanism for HF and has been called the “thrifty substrate” hypothesis [17, 25]. This hypothesis proposes that SGLT-2i induce ketogenesis, including β-hydroxybutyrate, in patients with T2DM [25, 26]. β-hydroxybutyrate is freely taken up by the heart and used as an energy-efficient “superfuel” to improve cardiac metabolism, such as oxygen consumption, at the mitochondrial level and also increase cardiac hydraulic efficiency in animal HF model [17, 18, 27, 28]. Finally, SGLT-2i may exert a cardioprotective effect by lowering uric acid level. Plasma uric acid level is associated with congestive HF and CVD [29–31]. SGLT-2i use causes secretion of uric acid via the GLUT9 transporter, resulting in a 10–15% reduction in plasma uric acid level [32, 33].

Previous RCTs included only patients with underlying CVD or patients at high risk for CVD; thus, there is a concern whether SGLT-2i have similar benefits in patients with T2DM who do not have established CVD. One observational study showed that SGLT-2i use on T2DM patients without underlying DM complication showed lower HR without statistical significance (adjusted HR = 0.83; 95% CI = 0.54–1.27; p = 0.40) [34]. However, in our study, hHF decreased significantly in the third year among patients without underlying CVD. The difference between our study result and that of Gautam et al. was probably due to the fact that we performed subgroup analysis according to the presence of underlying cardiovascular disease, whereas their analysis was based on the presence of any DM complications (any microvascular or macrovascular complications). Furthermore, they enrolled just 5000 patients, which was smaller than our study. To our knowledge, no observational study has analyzed the HF protective effect of SGLT-2i use with CVD stratification. This is the first study to estimate the HF protective effect among patients without established CVD.

In our study, SGLT-2i use reduced hHF compared with DPP-4i use beginning 30 days after initiating the drug among patients with underlying CVD, which was far earlier than patients without CVD. This result is in agreement with the results of a previous EMPA-REG sub study showing that SGLT-2i use was associated with a lower risk of hospitalization for HF from 360 to 1440 days after the drug initiation [35]. Also, although there was no statistical analysis to determine whether HF risk varies with follow-up period in the CANVAS program, early separation in the K–M curve of hHF after drug initiation was more prominent in the EMPA-REG OUTCOME trial, where all participants had previous history of CVD, compared to the CANVAS program that enrolled 34.4% participants who had no previous history of CVD [6, 8]. Our finding and the results from previous studies suggests that the effectiveness of HF protective mechanism with SGLT-2i use could be different depending on the patient’s underlying CVD. As our results and results of previous RCTs show, patients with T2DM and established CVD could benefit from SGLT-2i use beginning when the drug is initiated. Among the several mechanisms described above, such as a hemodynamic effect, and shift in fuel energetics, SGLT-2i could be more beneficial to patients with established CVD. One study showed that SGLT-2i decreased systolic blood pressure and increased diuresis among T2DM patients in just 48 h after initiation [20], and that this effect lasts relatively longer [22]. Also, after just a single dose of SGLT-2i, T2DM patients showed increased ketogenesis, which could directly benefit hemodynamics in heart failure patients [17, 25, 28]. Maybe these hemodynamic or metabolic changes could protect T2DM patients with established CVD immediately after SGLT-2i use, but later in patients without established CVD. However, our study did not analyze laboratory or echocardiographic results to determine the underlying mechanisms. Therefore, further evaluation is needed to identify the HF protective mechanism in patients with or without underlying CVD.

Several strengths of the present study should be mentioned. First, to our knowledge, this is the first study to estimate HF risk with CVD stratification and show that the HF protective effect of SGLT-2i can vary depending on whether patients have established CVD or not. Second, our results are the first to show that SGLT-2i have significant effects on HF beginning just 30 days after initiating the drug. This could be a meaningful finding and confirm the conjectures of previous studies. Third, this study was a nationwide population-based cohort study including over 50,000 new users of SGLT-2i. Finally, our study compared the SGLT-2i class, which is of great interest, with DPP-4i, one of the most widely used oral hypoglycemic agents in the world. By comparing these two drug classes and strictly adjusting all other oral hypoglycemic agents associated with hHF, physicians who consider prescribing both drugs will be directly helped in clinical situations with real world data.

Our research had some limitations. First, we used claims data, which do not contain information about measurements such as laboratory or echocardiography results, New York Heart Association Functional Classification of heart failure, socio-economic status, or diabetes duration; consequently, residual confounding factors probably existed. To compensate for this limitation, we performed propensity score matching with 55 variables including several complications of diabetes and HF medications. However, propensity score matching is not a remedy for all confounding factors, so our results should be interpreted with caution. In addition, we only adjusted prescribed medication before, but not after, the index date and, thus, there are still residual confounding factors. Second, we could not analyze mortality after use of SGLT-2i because the HIRA database does not contain mortality data. As previous RCTs and observational studies have already shown that SGLT-2i use is associated with improved mortality, our study could be more meaningful as it focused on the effect of SGLT-2i on HF in greater detail. Third, we could not analyze the HF risk of SGLT-2i according to the presence of underlying HF because there were only two thousand patients with underlying heart failure, which was too small for analysis.

## Conclusions

Our results suggest that SGLT-2i reduced hHF compared with DPP-4i. A HF protective effect of SGLT-2i vs. DPP-4i was shown 30 days after initiating the SGLT-2i among patients with established CVD, but this effect appeared later in patients without established CVD.

## Additional file

**Additional file 1: Table S1.** Baseline characteristics of all patients before propensity score matching. Data presented as frequencies in percentage or means (SD). *Confirmed by diagnosis code (International Classification of Diseases, 10th revision). *ACEI* angiotensin-converting-enzyme inhibitor, *AMI* acute myocardial infarction, *ARB* angiotensin II receptor antagonists, *DPP-4i* dipeptidyl-peptidase IV inhibitor, *NOAC* novel oral anticoagulant, *SD* standard deviation, *SGLT2i* sodium-glucose co-transporter 2 inhibitor, *SU* sulfonylurea. **Table S2.** Baseline characteristics of matched patients with established cardiovascular disease. Data presented as frequencies in percentage or means (SD). Less than 0.1 (10%) on the absolute value of standardized difference was considered as a negligible difference between groups. The mean (SD) standardized difference of all covariates was 1.01% (1.33%). *Confirmed by diagnosis code (International Classification of Diseases, 10th revision). *ACEI* angiotensin-converting-enzyme inhibitor, *AMI* acute myocardial infarction, *ARB* angiotensin II receptor antagonists, *DPP-4i* dipeptidyl-peptidase IV inhibitor, *NOAC* novel oral anticoagulant, *SD* standard deviation, *SGLT2i* sodium-glucose co-transporter 2 inhibitor, *SU* sulfonylurea. **Table S3.** Baseline characteristics of matched patients without established cardiovascular disease. Data presented as frequencies in percentage or means (SD). Less than 0.1 (10%) on the absolute value of standardized difference was considered as a negligible difference between groups. The mean (SD) standardized difference of all covariates was 0.96% (2.03%). *Confirmed by diagnosis code (International Classification of Diseases, 10th revision). *ACEI* angiotensin-converting-enzyme inhibitor, *ARB* angiotensin II receptor antagonists, *DPP-4i* dipeptidyl-peptidase IV inhibitor, *NOAC* novel oral anticoagulant, *SD* standard deviation, *SGLT2i* sodium-glucose co-transporter 2 inhibitor, *SU* sulfonylurea. The English in this document has been checked by at least two professional editors, both native speakers of English. For a certificate, please see: http://textcheck.com/certificate/index/dIr6Nw.

## Abbreviations

CANVAS: Canagliflozin Cardiovascular Assessment Study Program
CI: confidence interval
CVD: cardiovascular disease
DM: diabetes mellitus
DPP-4i: dipeptidyl peptidase-4 inhibitors
EMPA-REG OUTCOME: Empagliflozin Cardiovascular Outcome Event Trial in Type 2 Diabetes Mellitus Patients trial
HF: heart failure
hHF: hospitalization for heart failure
HR: hazard ratio
K–M: Kaplan–Meier
N: number
RCT: randomized controlled trial
SD: standard deviation
SGLT-2i: sodium glucose co-transporter 2 inhibitors
T2DM: type 2 diabetes mellitus
