# Supplementary material for: Association between sodium glucose co-transporter 2 inhibitors and a reduced risk of heart failure in patients with type 2 diabetes mellitus: a real-world nationwide population-based cohort study
Source: Cardiovasc Diabetol. 2018 Jun 23;17:91. doi: 10.1186/s12933-018-0737-5 (PMC6015464; doi:10.1186/s12933-018-0737-5)
Supplement: Supplementary file 1 — Additional file 1: Table S1. Baseline characteristics of all patients before propensity score matching. Data presented as frequencies in percentage or means (SD). *Confirmed by diagnosis code (International Classification of Diseases, 10th revision). ACEI angiotensin-converting-enzyme inhibitor, AMI acute myocardial infarction, ARB angiotensin II receptor antagonists, DPP-4i dipeptidyl-peptidase IV inhibitor, NOAC novel oral anticoagulant, SD standard deviation, SGLT2i sodium-glucose co-transporter 2 inhibitor, SU sulfonylurea. Table S2. Baseline characteristics of matched patients with established cardiovascular disease. Data presented as frequencies in percentage or means (SD). Less than 0.1 (10%) on the absolute value of standardized difference was considered as a negligible difference between groups. The mean (SD) standardized difference of all covariates was 1.01% (1.33%). *Confirmed by diagnosis code (International Classification of Diseases, 10th revision). ACEI angiotensin-converting-enzyme inhibitor, AMI acute myocardial infarction, ARB angiotensin II receptor antagonists, DPP-4i dipeptidyl-peptidase IV inhibitor, NOAC novel oral anticoagulant, SD standard deviation, SGLT2i sodium-glucose co-transporter 2 inhibitor, SU sulfonylurea. Table S3. Baseline characteristics of matched patients without established cardiovascular disease. Data presented as frequencies in percentage or means (SD). Less than 0.1 (10%) on the absolute value of standardized difference was considered as a negligible difference between groups. The mean (SD) standardized difference of all covariates was 0.96% (2.03%). *Confirmed by diagnosis code (International Classification of Diseases, 10th revision). ACEI angiotensin-converting-enzyme inhibitor, ARB angiotensin II receptor antagonists, DPP-4i dipeptidyl-peptidase IV inhibitor, NOAC novel oral anticoagulant, SD standard deviation, SGLT2i sodium-glucose co-transporter 2 inhibitor, SU sulfonylurea. The English in this document has been ch [file 12933_2018_737_MOESM1_ESM.docx]

**Additional Materials**

**Additional file 1: Table S1. Baseline characteristics of all patients before propensity score matching.**

|  | DPP-4i  ( n = 1,044,194 ) | SGLT-2i  ( n = 59,480 ) | Standardized difference |
| --- | --- | --- | --- |
| Age (years), mean (SD) | 58.0 (11.6) | 53.2 (11.9) | 0.406 |
| Male | 59.50 | 54.86 | 0.094 |
| Hypertension | 59.69 | 60.33 | 0.013 |
| Dyslipidemia | 76.50 | 82.05 | 0.137 |
| Chronic kidney disease | 7.21 | 7.55 | 0.013 |
| Cardiovascular disease |  |  |  |
| Acute myocardial infarction | 1.39 | 1.43 | 0.003 |
| Other ischemic heart disease | 12.90 | 14.02 | 0.033 |
| Heart failure | 3.21 | 3.41 | 0.011 |
| Cerebral infarction | 5.89 | 3.71 | 0.102 |
| Cerebrovascular event | 7.62 | 5.24 | 0.097 |
| Peripheral artery occlusive disease | 0.84 | 0.61 | 0.027 |
| Coronary revascularization procedures |  |  |  |
| Coronary artery bypass graft | 0.06 | 0.03 | 0.014 |
| Percutaneous coronary intervention | 1.91 | 1.99 | 0.006 |
| Microvascular complications of diabetes |  |  |  |
| Neuropathy | 10.97 | 10.34 | 0.030 |
| Nephropathy | 6.26 | 6.99 | 0.020 |
| Retinopathy | 13.28 | 12.93 | 0.011 |
| Atrial fibrillation | 1.86 | 1.52 | 0.026 |
| Other heart disease | 10.38 | 11.71 | 0.042 |
| Hypoglycemia | 2.30 | 2.16 | 0.010 |
| Asthma | 12.36 | 12.49 | 0.004 |
| Chronic obstructive pulmonary disease | 5.65 | 4.74 | 0.041 |
| Connective tissue disease | 3.73 | 3.58 | 0.008 |
| Pancreatitis | 1.49 | 1.49 | <0.001 |
| Osteoporosis | 9.66 | 7.36 | 0.083 |
| Alcohol intake^*^ | 5.10 | 4.65 | 0.021 |
| Smoking^*^ | 0.10 | 0.16 | 0.017 |
| Obesity^*^ | 0.312 | 0.39 | 0.054 |
| Medication use (180 days prior to index date) |  |  |  |
| Antidiabetic agent |  |  |  |
| Metformin | 92.68 | 81.18 | 0.346 |
| Sulfonylurea | 46.82 | 36.77 | 0.205 |
| Thiazolidinediones | 8.27 | 9.11 | 0.030 |
| Alpha-glucosidase inhibitor | 6.29 | 4.71 | 0.069 |
| Meglitinide | 0.97 | 0.73 | 0.026 |
| Insulin | 14.41 | 13.84 | 0.016 |
| Steroid | 33.71 | 34.60 | 0.019 |
| Diuretics |  |  |  |
| Loop diuretics | 5.05 | 3.79 | 0.061 |
| Thiazide | 15.14 | 14.68 | 0.013 |
| Aldosterone antagonist | 2.14 | 1.92 | 0.016 |
| Potassium sparing diuretics | 0.09 | 0.06 | 0.008 |
| Anti-hypertensive agent |  |  |  |
| Calcium channel blocker | 30.18 | 29.74 | 0.010 |
| Angiotensin-converting-enzyme inhibitor | 3.04 | 2.95 | 0.005 |
| Angiotensin II receptor antagonists | 45.50 | 47.62 | 0.042 |
| Beta blocker | 9.35 | 9.65 | 0.010 |
| Alpha blocker | 0.96 | 0.67 | 0.032 |
| Digoxin | 1.03 | 0.72 | 0.033 |
| Aspirin | 26.79 | 22.98 | 0.088 |
| P2Y12 inhibitor | 8.84 | 8.38 | 0.016 |
| Warfarin | 0.93 | 0.58 | 0.041 |
| Novel oral anticoagulant | 0.62 | 0.53 | 0.011 |
| Lipid-lowering agent |  |  |  |
| Statin | 49.76 | 53.02 | 0.065 |
| Fibrate | 8.86 | 10.74 | 0.063 |
| Ezetimibe | 5.52 | 8.51 | 0.117 |
| Cardiologist visit (30 days prior to index date) | 10.01 | 12.40 | 0.076 |
| Hospitalization (30 days prior to index date) | 12.51 | 6.90 | 0.190 |
| Hospitalization (30-365days prior to index date) | 25.12 | 20.30 | 0.115 |
| Emergency department visit (365 days prior to index date) | 8.24 | 5.84 | 0.094 |

Data presented as frequencies in percentage or means (SD).

* Confirmed by diagnosis code (International Classification of Diseases, 10^th^ revision)

ACEI, angiotensin-converting-enzyme inhibitor; AMI, acute myocardial infarction; ARB, angiotensin II receptor antagonists; DPP-4i, dipeptidyl-peptidase IV inhibitor; NOAC, novel oral anticoagulant; SD, standard deviation; SGLT2i, sodium-glucose co-transporter 2 inhibitor; SU, sulfonylurea.

**Additional file 1: Table S2. Baseline characteristics of matched patients with established cardiovascular disease.**

|  | DPP-4i  ( n = 11,188 ) | SGLT-2i  ( n = 11,188 ) | Standardized difference |
| --- | --- | --- | --- |
| Age (years), mean (SD) | 59.7 (10.8) | 59.7 (10.3) | 0.003 |
| Male | 57.61 | 57.18 | 0.009 |
| Hypertension | 84.9 | 84.72 | 0.005 |
| Dyslipidemia | 92.06 | 92.07 | <0.001 |
| Chronic kidney disease | 10.43 | 10.66 | 0.008 |
| Cardiovascular disease | 0 | 0 |  |
| AMI | 7.46 | 7.62 | 0.006 |
| Other ischemic heart disease | 74.07 | 74.53 | 0.01 |
| Heart failure | 11.1 | 11.3 | 0.006 |
| Cerebral infarction | 20.07 | 19.74 | 0.008 |
| Cerebrovascular event | 28.18 | 27.88 | 0.007 |
| Peripheral artery occlusive disease | 3.3 | 3.25 | 0.003 |
| Coronary revascularization procedures | 0 | 0 |  |
| Coronary artery bypass graft | 0.17 | 0.18 | 0.002 |
| Percutaneous coronary intervention | 10.39 | 10.59 | 0.007 |
| Microvascular complications of diabetes | 0 | 0 |  |
| Nephropathy | 14.77 | 14.33 | 0.013 |
| Neuropathy | 9.45 | 9.89 | 0.015 |
| Retinopathy | 18.3 | 18.7 | 0.01 |
| Atrial fibrillation | 4.77 | 4.7 | 0.003 |
| Other heart disease | 25.14 | 25.28 | 0.003 |
| Hypoglycemia | 2.75 | 2.74 | 0.001 |
| Asthma | 15.97 | 15.98 | <0.001 |
| Chronic obstructive pulmonary disease | 7.94 | 7.95 | 0.001 |
| Connective tissue disease | 4.81 | 4.88 | 0.003 |
| Pancreatitis | 2.32 | 2.4 | 0.005 |
| Osteoporosis | 11.61 | 11.77 | 0.005 |
| Alcohol intake* | 3.83 | 4.13 | 0.016 |
| Smoking* | 0.55 | 0.55 | <0.001 |
| Obesity* | 0.39 | 0.29 | 0.017 |
| Medication use (180 days prior to index date) | 0 | 0 |  |
| Antidiabetic agent | 0 | 0 |  |
| Metformin | 79.72 | 79.84 | 0.003 |
| Sulfonylurea | 41.74 | 41.32 | 0.009 |
| Thiazolidinediones | 10.57 | 10.29 | 0.009 |
| Alpha-glucosidase inhibitor | 6.34 | 5.99 | 0.014 |
| Meglitinide | 1.09 | 1.04 | 0.005 |
| Insulin | 20.5 | 20.7 | 0.005 |
| Steroid | 35.98 | 35.95 | 0.001 |
| Diuretics | 0 | 0 |  |
| Loop diuretics | 9.98 | 9.67 | 0.011 |
| Thiazide | 17.88 | 18.1 | 0.006 |
| Aldosterone antagonist | 4.71 | 4.7 | <0.001 |
| Potassium sparing diuretics | 0.1 | 0.13 | 0.008 |
| Anti-hypertensive agent | 0 | 0 |  |
| Calcium channel blocker | 39.19 | 39.29 | 0.002 |
| ACEI | 8.41 | 8.1 | 0.011 |
| ARB | 63.48 | 63.44 | 0.001 |
| Beta blocker | 25.24 | 25.06 | 0.004 |
| Alpha blocker | 1.16 | 1.21 | 0.004 |
| Digoxin | 2.16 | 2.07 | 0.006 |
| Aspirin | 56.77 | 56.02 | 0.015 |
| P2Y12 inhibitor | 37.6 | 37.75 | 0.003 |
| Warfarin | 1.84 | 1.78 | 0.005 |
| NOAC | 1.73 | 1.64 | 0.006 |
| Lipid-lowering agent |  |  |  |
| Statin | 71.72 | 71.59 | 0.003 |
| Fibrate | 9.86 | 10.06 | 0.007 |
| Ezetimibe | 9.57 | 10.03 | 0.015 |
| Cardiologist visit (30 days prior to index date) | 42.03 | 42.5 | 0.01 |
| Hospitalization (30 days prior to index date) | 11.79 | 11.89 | 0.003 |
| Hospitalization (30-365days prior to index date) | 33.18 | 33.49 | 0.007 |
| Emergency department visit (365 days prior to index date) | 10.43 | 10.71 | 0.009 |

Data presented as frequencies in percentage or means (SD).

Less than 0.1 (10%) on the absolute value of standardized difference was considered as a negligible difference between groups. The mean (SD) standardized difference of all covariates was 1.01% (1.33%).

* Confirmed by diagnosis code (International Classification of Diseases, 10^th^ revision)

ACEI, angiotensin-converting-enzyme inhibitor; AMI, acute myocardial infarction; ARB, angiotensin II receptor antagonists; DPP-4i, dipeptidyl-peptidase IV inhibitor; NOAC, novel oral anticoagulant; SD, standard deviation; SGLT2i, sodium-glucose co-transporter 2 inhibitor; SU, sulfonylurea.

**Additional file 1: Table S3. Baseline characteristics of matched patients without established cardiovascular disease.**

|  | DPP-4i  ( n = 48,290 ) | SGLT-2i  ( n = 48,290 ) | Standardized difference |
| --- | --- | --- | --- |
| Age (years), mean (SD) | 51.9 (12.1) | 51.7 (11.7) | 0.013 |
| Male | 54.33 | 54.33 | <0.001 |
| Hypertension | 55 | 54.68 | 0.006 |
| Dyslipidemia | 79.43 | 79.73 | 0.007 |
| Chronic kidney disease | 7 | 6.83 | 0.007 |
| Microvascular complications of diabetes |  |  |  |
| Nephropathy | 9.42 | 9.42 | <0.001 |
| Neuropathy | 6.49 | 6.32 | 0.007 |
| Retinopathy | 11.87 | 11.59 | 0.009 |
| Atrial fibrillation | 0.83 | 0.79 | 0.004 |
| Other heart disease | 8.52 | 8.57 | 0.002 |
| Hypoglycemia | 2.07 | 2.03 | 0.003 |
| Asthma | 11.79 | 11.68 | 0.003 |
| Chronic obstructive pulmonary disease | 4.01 | 3.99 | 0.001 |
| Connective tissue disease | 3.23 | 3.28 | 0.003 |
| Pancreatitis | 1.31 | 1.27 | 0.003 |
| Osteoporosis | 6.49 | 6.33 | 0.006 |
| Alcohol intake* | 4.73 | 4.78 | 0.002 |
| Smoking* | 0.05 | 0.07 | 0.007 |
| Obesity* | 0.43 | 0.41 | 0.004 |
| Medication use (180 days prior to index date) |  |  |  |
| Antidiabetic agent |  |  |  |
| Metformin | 81.22 | 81.5 | 0.007 |
| Sulfonylurea | 36.2 | 35.72 | 0.01 |
| Thiazolidinediones | 9.04 | 8.84 | 0.007 |
| Alpha-glucosidase inhibitor | 4.63 | 4.42 | 0.01 |
| Meglitinide | 0.69 | 0.66 | 0.004 |
| Insulin | 12.4 | 12.26 | 0.004 |
| Steroid | 34.62 | 34.28 | 0.007 |
| Diuretics |  |  |  |
| Loop diuretics | 2.42 | 2.43 | 0.001 |
| Thiazide | 13.85 | 13.89 | 0.001 |
| Aldosterone antagonist | 1.23 | 1.28 | 0.005 |
| Potassium sparing diuretics | 0.05 | 0.05 | 0.001 |
| Anti-hypertensive agent |  |  |  |
| Calcium channel blocker | 27.8 | 27.52 | 0.006 |
| ACEI | 1.75 | 1.76 | 0.001 |
| ARB | 44.23 | 43.96 | 0.005 |
| Beta blocker | 6.29 | 6.08 | 0.009 |
| Alpha blocker | 0.55 | 0.54 | <0.001 |
| Digoxin | 0.41 | 0.41 | <0.001 |
| Aspirin | 15.44 | 15.33 | 0.003 |
| P2Y12 inhibitor | 1.66 | 1.57 | 0.007 |
| Warfarin | 0.31 | 0.3 | 0.002 |
| NOAC | 0.28 | 0.27 | 0.001 |
| Lipid-lowering agent |  |  |  |
| Statin | 48.66 | 48.72 | 0.001 |
| Fibrate | 10.82 | 10.9 | 0.003 |
| Ezetimibe | 8.02 | 8.16 | 0.005 |
| Cardiologist visit (30 days prior to index date) | 5.48 | 5.43 | 0.002 |
| Hospitalization (30 days prior to index date) | 5.88 | 5.74 | 0.006 |
| Hospitalization (30-365days prior to index date) | 17.42 | 17.25 | 0.005 |
| Emergency department visit (365 days prior to index date) | 4.66 | 4.71 | 0.003 |

Data presented as frequencies in percentage or means (SD).

Less than 0.1 (10%) on the absolute value of standardized difference was considered as a negligible difference between groups. The mean (SD) standardized difference of all covariates was 0.96% (2.03%).

* Confirmed by diagnosis code (International Classification of Diseases, 10^th^ revision)

ACEI, angiotensin-converting-enzyme inhibitor; ARB, angiotensin II receptor antagonists; DPP-4i, dipeptidyl-peptidase IV inhibitor; NOAC, novel oral anticoagulant; SD, standard deviation; SGLT2i, sodium-glucose co-transporter 2 inhibitor; SU, sulfonylurea.
